# P-1116. Another One Bites the Flush: Metagenomic Next-Generation Sequencing (mNGS) Signal leads to Rapid Water Safety Overhaul on a Bone Marrow Transplant (BMT) Unit

**DOI:** 10.1093/ofid/ofaf695.1311

**Published:** 2026-01-11

**Authors:** Ioana Chirca, Carrigan Hayes, Leigh Ann Kelly, Grace Lai, Scott Sukits, Nathan Hyatt, Steven Concepcion, Delrico De Guzman, Katrina Vargas Mesa, Robert Koger, Jose Alexander, Vincent Hsu

**Affiliations:** AdventHealth, Evans, GA; AdventHealth, Evans, GA; AdventHealth, Evans, GA; AdventHealth, Evans, GA; AdventHealth, Evans, GA; AdventHealth, Evans, GA; AdventHealth, Evans, GA; AdventHealth, Evans, GA; AdventHealth, Evans, GA; AdventHealth, Evans, GA; Advent Health Central Florida, Orlando, FL; AdventHealth, Evans, GA

## Abstract

**Background:**

Hospital-onset legionellosis is difficult to control as *Legionella* thrives inside pluming biofilms, evades routine culturing methods and disproportionately affects immunocompromised patients. A water management (WM) program is a regulatory requirement. Turning genomic signals into immediate, unit-wide controls remains under-reported.

**Methods:**

Two neutropenic patients in a BMT unit of a 1400-bed tertiary hospital were diagnosed with *L. dumoffii* pneumonia via cell-free mNGS within a week of each other. A multidisciplinary incident command activated the WM plan that included: closure of rooms that housed the two patients during the index hospitalization and patient relocation, bottled water for consumption and oral care, suspension of showering, and unit-wide vigorous flushing of every fixture. Environmental sampling included all fixtures in the rooms that housed the patients, other selected outlets on the unit, and all ice machines. Positive outlets received 10 % chlorine disinfection. Daily flushing with audit logs, monthly fixture disinfection, enhanced water temperature and residual oxidant audits, removal of ice machines, a 3-ft sink clutter-free zone, and an ANC-based shower restriction were added.

**Results:**

Of 22 water samples, three fixtures in the index room yielded *Legionella* species which were further speciated by 16S tNGS as *L. dumoffii*. The whole genome sequencing and ribosomal multilocus sequence type (rMLST) of these isolates, performed at the hospital’s laboratory, demonstrated related strains NY-23 and rMLST 39551 on all three isolates. Water temperature and residual oxidant values were within expected parameters and daily flushing compliance reached 100%. Water flushing declinations were resolved. No additional *Legionella* cases were detected during 90-day enhanced surveillance. Both patients fully recovered.

**Conclusion:**

Rapid mNGS diagnosis, followed by a policy-driven, multidisciplinary response, rapidly contained a *L. dumoffii* cluster and prevented further cases. This genomics-to-policy approach, integrating advanced diagnostics, engineering controls, and patient-specific precautions can safeguard immunocompromised populations.

**Disclosures:**

All Authors: No reported disclosures

